# Multiracial individuals’ perspectives on participating in genetics research

**DOI:** 10.1007/s12687-026-00902-x

**Published:** 2026-06-05

**Authors:** Emilia Chiriboga, Christin Hoell, Daphne Oluwaseun Martschenko, Genevieve L. Wojcik, Hannah Wand, Jennifer L. Young

**Affiliations:** 1 Graduate Program in Genetic Counseling, Northwestern University, Chicago, IL USA; 2 Laurie J. Girand Center for Biomedical Ethics, Department of Pediatrics, Stanford University School of Medicine, Stanford, CA USA; 3 Bloomberg School of Public Health, Johns Hopkins University, Baltimore, MD USA; 4 Center for Inherited Cardiovascular Disease & Center for Undiagnosed Diseases, Stanford University, Palo Alto, CA USA; 5 Department of Medicine, Biological Sciences Division, The University of Chicago, Chicago, IL USA

**Keywords:** Multiracial, Community Engagement, Research Translation, Genetic, Genomic

## Abstract

The vast majority of genetics research is confined to a relatively narrow subset of the global population, limiting the benefits of this research. Recommendations to ameliorate this issue frequently include calls to recruit diverse populations through community engagement, an approach that has been effective in genetics, but has not explicitly included Multiracial individuals, despite this being the fastest-growing population in the United States. As such, this study explored Multiracial individuals’ perspectives on: (1) what “Multiracial community” means; (2) concerns regarding ancestry-based genetic testing; and (3) preferences for engaging in different stages of translational genetics research. Fourteen adults who self-identified as Multiracial participated in semi-structured interviews. We used deductive coding from study aims and inductive, iterative coding to identify new themes. Participants expressed diverse ideas about what characterizes the Multiracial community, and lacked clarity on definitions. Common concerns about genetics research included the accuracy of genetics research in its application for Multiracial individuals and the limitations of research to capture their diverse backgrounds. Participants preferred to be engaged during research design, recruitment, and result interpretation and communication. Participants suggested recruiting future study participants from younger, urban regions and emphasized that this is a multifaceted heterogeneous group that cannot easily be categorized. We describe the perspectives of Multiracial individuals, who are often excluded from genetics research because their diverse social identities do not fit traditional methods of categorization. This provides a foundation for developing tailored approaches to recruit and engage Multiracial participants in genetics research, ensuring future studies better reflect human diversity.

**Supplementary Information:**

The online version contains supplementary material available at 10.1007/s12687-026-00902-x.

## Introduction

The vast majority of genetics research has led to findings that only benefit a small portion of the global population. As such, scholars have warned that clinical applications of genomic research could exacerbate health disparities (Bentley et al. 2017). This issue is the result of logistical, systemic, and historical factors in the research process (Popejoy and Fullerton 2016). While many have historically been excluded from genetics research, Multiracial individuals are notably absent. Yet, Multiracial individuals comprise the fastest growing racial group in the United States; the most recent Census revealed a significant increase in the number of individuals who identify as Multiracial – from 9 million in 2010 to 33.8 million in 2020, a 276% increase (Jones 2021). While this may partly capture a difference in reporting, this increase is consistent with a broader trend of growing numbers of Multiracial individuals (Parker et al. 2015; Starr 2024).

Multiracial individuals have been excluded from scientific research for a variety of sociopolitical factors that shape the research process (Martschenko 2022). For example, until the 1970 s, the United States practiced hypodescent, or the “one drop rule”, as people born to European and African parents were legally required to identify as Black (Deters 1997). Over time, progress has been made to acknowledge the existence of Multiracial identities in the US. In 2000, the US Census allowed individuals to self-identify with multiple races for the first time (Jones 2021). Nonetheless, categorizing people into discrete racial and ethnic groups remains in both science and society. Multiracial individuals may still find themselves forced to select one out of many racial categories or to select broad, encompassing terms such as “other”. They may also feel social pressures to affiliate with a single racial group depending upon the context. As a result, Multiracial individual’s unique identities are often missing (Deters 1997). Driving this disparity even further is the fact that classic statistical methods in genetics are not always accurate or applicable for the populations that were excluded when these methodologies were created (Korunes and Goldberg 2021). This frequently results in the omission of data from Multiracial participants when analyzing health research findings, contributing to the lack of information that exists on this community today (Martschenko et al. 2023).

Recently, community engagement has emerged as an intervention for diversifying research, particularly in genetics (NASEM 2024). Community engagement entails working collaboratively with groups of people to build trust, broaden the benefits of research, increase participation, and identify areas of commonality (CDC 1997; Lemke et al. 2022; Holzer et al. 2014). A meta-analysis focused on the effectiveness of utilizing community engagement as a tool in public health interventions for disadvantaged groups demonstrated that community-engaged research has a positive impact on a range of health and psychosocial outcomes (O’Mara-Eves et al. 2015). Similarly, community members involved in community-engaged research have acknowledged the benefits that result from utilizing this approach, especially when mutual trust and transparency are promoted (Han et al. 2021). This approach has also been used in genomics research - one study explored the viewpoints of African American individuals on *APOL1* testing and reached mutually agreed upon recommendations by continuously including community members (Young et al. 2019). Similarly, a study involving individuals from the American Indian and Alaska Native communities was able to identify unique potential barriers (e.g., opportunity cost and misuses of genetic information leading to stigmatization) and facilitators (e.g., strong and effective community oversight of research and clinical care) through continuous discussions with community members (Woodahl et al. 2014). Although these studies and others have shown that community engagement in healthcare research is often feasible and beneficial for the target community, it is crucial to ensure that this involvement does not become merely tokenistic (Domecq et al. 2014).

Despite strong evidence supporting the utility and benefit of employing community-engaged research in healthcare research, to our knowledge no empirical studies have examined the utility of this approach when it comes to involving Multiracial individuals, specifically within the realm of genetics research. To date, the majority of research on Multiracial individuals has been confined to the fields of psychology, sociology, social work, education, and public health (Charmaraman et al. 2015), leading to a limited understanding of the needs and preferences of this population within the field of genetics. Current exclusionary practices, on top of a lack of guidelines for how to define, recruit, and engage Multiracial individuals, pose serious implications for the future of research involving this community (Martschenko et al. 2023).

This study adds empirical data by qualitatively exploring Multiracial individuals’ perspectives on: (1) their inclusion in genetics research; (2) their needs, desires, and preferences in engaging with the translational research process; and (3) best methods for recruiting this diverse population into research. For the purposes of this study, we define Multiracial identity as either having two biological parents who self-identify with different monoracial backgrounds, or at least one parent who identifies as Multiracial (Atkin 2023).While there is no genetic basis for race, which is a socially constructed identity, we acknowledge that sometimes race, ethnicity, and ancestry are used interchangeably by researchers and some of the participants in this study (Caliebe et al. 2022; NASEM 2024).We hope that shedding light on the perspectives of members of this community will inform researchers and guide more inclusive studies moving forward.

## Materials and methods

### Study design

This study employed semi-structured interviews to explore Multiracial individuals’ perspectives on genetics research. Each participant attended a single session lasting approximately one hour. The study was approved with exempt status following review by the Northwestern Institutional Review Board (STU00220221).

A semi-structured interview guide (Supplementary Item 1) was constructed by the research team, which included four Multiracial researchers (see Table 1 for positionality statements). The guide was designed to explore three distinct yet interconnected concepts: Multiracial identity, Multiracial community, and Multiracial engagement in genetics research. The guide was intentionally organized to progress from more familiar and specific topics to broader, more abstract concepts. This approach was chosen to ease participants into the discussion, allowing them to engage comfortably before delving into more complex, and perhaps unfamiliar subject matters.

**Table 1:** Positionality statements

**Emilia Chiriboga** (*she/her*): I identify as Hispanic, specifically Ecuadorian. The first half of my childhood was spent surrounded by my tight-knit family in Quito. The second half was spent in the United States, fortunately realizing my parents' dreams of providing my sister and me with more opportunities. Growing up in two distinct countries not only established my multicultural identity, but also led me to develop a passion for learning about and celebrating other cultures and traditions.	
**Christin Hoell ***(she/her)*: I identify as white and my family has lived in the United States for multiple generations, originally immigrating from Germany. I grew up in a community that was predominantly white and middle class, which led to a lack of recognition of the ways discrimination and systemic racism present themselves in today’s society. As I moved out of that community and became aware of my own privilege, I have aspired to use my platform as an educator to shine light on these issues and support my peers and students in their work.	
**Daphne O. Martschenko** *(she/her)*: I self-identify as a biracial African American and I am most often identified by others as Black. My parents both immigrated to the United States – my father as a child from the Ukraine and my mother as an adult from Nigeria. I was born in England and spent portions of my childhood living in various Eastern European countries, ever aware of the curiosity my biracial family sparked in passersby. My multicultural upbringing and challenges with my racial identity motivate my work investigating the social and ethical implications of genetic/genomics and the fraught and violent relationship between race, genetics, and human behavior.	
**Genevieve L. Wojcik** *(she/her)*: I am unsure as to how I identify, as my experience as a biracial individual in the United States has largely been defined by what I am not, instead of what I am. My mother immigrated here from Taiwan and my father’s parents from France and Poland. My research interests in genetic epidemiology for diverse, and specifically admixed populations, have been partially motivated by my background to ensure that discoveries will also benefit my loved ones, whether family or friends, with increased urgency for my multiracial children.	
**Hannah Wand** *(she/her)*: I identify as a biracial Asian American. While my family has direct physical ties to Korea and Poland, our biological family is small and we don’t have a strong sense of multigenerational lineage or culture, which further complicates my sense of racial identity. This complexity is something I’ve learned to embrace and even celebrate with time and life experience. Professionally, I draw on this ambiguous sense of self in my research on and advocacy for inclusive healthcare design.	
**Jennifer L. Young** *(she/her)*: I identify as a biracial Asian American woman, and I always explained my racial identity by sharing where my parents grew up. My father was from London, England and my mother was from Beijing, China. I grew up in a very international neighborhood in a midwestern United States college town, which exposed me to a range of different cultures, but people who identified as Multiracial were still a significant minority in my hometown. Issues of racial identity and Multiracial families has been at the center of my family systems research as well as my clinical work as a family therapist.	

The questions regarding Multiracial identity and community were predominantly open-ended, allowing participants to freely express their thoughts and experiences. Following this segment, participants were provided with a brief overview of the research process and presented with an example of genetics research on adrenocortical carcinoma cases in Southern Brazil (Achatz and Zambetti 2016). This case was chosen as it demonstrates a clear example of a high frequency variant within a specific population. After reviewing this example, participants were asked to consider the potential benefits or concerns of genetics research for individuals from Multiracial backgrounds. Then, they were presented with seven roles individuals can undertake during the research process: developing the research question, determining recruitment, defining the roles, collecting data, interpreting results, communicating findings, and determining compensation. These roles were inspired by recommendations published in a guidance document by Lemke et al., which focused on providing genetics and genomics researchers with strategies for working with marginalized and underrepresented communities (2022). The roles were strategically selected to span the entirety of the research cycle, capturing a more comprehensive view of the steps required to complete a research study, from study design to research communication. Participants were asked to rank the three roles they considered most important for them to provide their perspective as a Multiracial individual. Lastly, participants were asked to suggest recruitment strategies that could benefit future researchers seeking to engage Multiracial individuals, particularly regarding location and participant characteristics.

### Recruitment

Individuals who met the following eligibility criteria were recruited for this study: 18 years of age or older, identified as Multiracial, spoke English, resided in the United States, and had access to Zoom video conferencing software. Upon receiving approval from the Northwestern IRB, a study flier and screening survey were shared within the “Mixed Race Studies” Facebook group, a national group consisting of 8.5 K members. To boost recruitment numbers, the study announcement was subsequently posted in additional Facebook groups targeted towards Mixed race or biracial individuals and families, as well as groups that facilitate connections between researchers and community members. After completing a screening survey, eligible participants were sent a consent form via email. Those who returned the signed consent form were emailed an additional survey to collect demographic information and schedule interviews.

### Data collection

Interviews were held between January 2024 and February 2024 and audio-recorded via Zoom. Recruitment efforts concluded once the authors determined that data saturation had been reached (i.e., sufficient data was collected to validate prominent themes). Audio recordings were transcribed verbatim and de-identified by E.C. To compensate them for their time, participants were mailed a $40 VISA gift card.

### Data analysis

All transcripts were uploaded to Dedoose, a qualitative software program (Version 9.2.006). The data analysis utilized a combination of constructivist grounded theory and interpretive description (Dedoose 2021; Tie et al. 2019; Burdine et al. 2020). These approaches were chosen to ensure that participants’ perspectives and experiences were meaningfully captured, resulting in themes that were both detailed and representative.

The codebook was developed using both deductive and inductive approaches. Deductive content analysis relies on existing knowledge, whereas inductive content analysis is used when exploring new topics (Elo and Kyngäs 2008). In this study, the aims and semi-structured interview guide provided the “previous knowledge” for developing structural codes. Next, open coding was applied using an iterative memoing process on the transcripts (Morgan and Nica 2020). To test the codebook, two coders, E.C. and J.Y., independently coded three transcripts, discussed discrepancies, and made adjustments to the codebook. After reaching consensus on the precision of codes and definitions, E.C. independently coded the remaining transcripts. A third author, C.H., completed an inter-rater reliability test, which resulted in a pooled Cohen’s Kappa score of 1.0 (McHugh 2012).^1^

Preliminary themes were developed by E.C. and J.Y. using coding reports, memoing, and sensitizing concepts. These themes were discussed with C.H., D.O.M., and G.W. who assisted in clarifying and finalizing overarching themes, patterns, and perspectives within the data (Thompson 2022).

## Results

### Participant characteristics

We conducted a total of 11 interviews involving 14 participants. Three interviews included two participants each, while the remaining eight were individual interviews. . Participant demographics are presented in Table 2 and their self-described racial identities, as provided on the screening survey, are presented in Table 3. Participants were located in different geographic areas across the US: two from the West, three from the Northeast, four from the South, and five from the Midwest. There were equal numbers of men and women represented in the sample, with an age range of 18–38 years old. The highest level of education achieved among participants varied, with most participants (*n* = 9) having received a Bachelor’s degree or higher. Income levels also varied, with a little over half of individuals (*n* = 8) reporting an annual income of less than $50,000. Notably, four participants were employed in healthcare, and one participant was undergoing training to join this field.

**Table 2:** Participant demographics

Gender	*N*
Female	7
Male	7
**Age**	**N**
Range	18–38 years
Mean	27 years
**Highest Level of Education**	**N**
Some High School	1
High School	2
Trade or Vocational School	2
Bachelor’s Degree	5
Master’s Degree	3
Doctorate	1
**Income**	**N**
Less than $20,000	5
$35,000 to $49,999	3
$50,000 to $74,999	2
$75,000 to $99,999	1
Over $100,000	3

**Table 3:** Multiracial identities

ID	Race/Ethnicity
1	Black, White, and Indigenous
2	Black (Liberian) and Middle Eastern (Saudi Arabian)
3	Middle Eastern and White
4	Filipino and White
5	Afro-Latino and White
6	Middle Eastern (Yemeni) and Mixed-Race (Javanese and Thai)
7	Black and White
8	Asian (Filipino and Chinese) and White (Irish and German)
9	Hispanic (Colombian) and White (Irish and German)
10	Middle Eastern (Persian) and White (European)
11	Hispanic (Argentinian) and White
12	Filipino and White
13	Asian (Indonesian) and White
14	Hispanic (Chilean) and White

### Themes

Four primary themes emerged from this study. The first theme describes participants’ perceptions of the Multiracial community. The second theme sheds light on participants’ concerns regarding genetics research involving Multiracial individuals. The third theme describes participants’ preferences for engaging with different stages of the translational research process. Lastly, the fourth theme highlights suggestions made by participants for recruiting individuals from the Multiracial population to inform future studies involving this community.

### Theme 1: Evolving definition of “Multiracial Community”

Participants were asked to consider whether they perceive a distinct Multiracial community, and if so, what characterizes this community from others, and what their experiences have been in this community. Participants expressed a wide range of ideas regarding the concept of a Multiracial community, reflecting the diverse range of perspectives within this population. The viewpoints shared by participants can be broken down into three separate response types.

First, some participants (*n* = 5) shared the belief that a community of Multiracial individuals does not actually exist. Individuals who held this belief often cited the lack of diversity within their own city or the region where they grew up, which led them to have limited interactions with other Multiracial individuals. Participant 10 was among the individuals with this viewpoint, as evidenced by the following quote:

*“I don’t think there’s a specific community where it’s like. Alright I’m half and half and you’re half and half*,* so therefore let’s hang out with each other for that reason*,* no” (Participant 10*,* Middle Eastern & White)*.

While this participant rejected the existence of a Multiracial community, he recognized that individuals from diverse backgrounds may be naturally drawn to each other more often. He pointed out that this tendency is typically “subconscious”, reinforcing his belief that a community composed of Multiracial individuals is not deliberately formed.

Second, a few participants (*n* = 3) drew on their personal experiences to characterize the Multiracial community, illustrating how their unique backgrounds directly influence their perceptions. More specifically, these participants referenced their own Multiracial identity or the composition of their family when defining the Multiracial community. For instance, Participant 12, who identifies as Filipino and White, noted that for him, the Multiracial community exists within his friend group which, like him, consists of individuals who identify as both White and Asian. Another participant, Participant 7, stated that when he thinks of the Multiracial community, he thinks of “anybody who is mixed race, like [his] own family”, demonstrating how his Black and White heritage influences his viewpoint. Notably, all participants who drew on their personal background to define the Multiracial community identified as belonging to exactly two racial groups (i.e., Biracial), rather than three or more (i.e., Multiracial).

*“When I think about like a Multiracial community*,* I would say I typically think of it as like people who have a Hispanic parent and a White parent. Just because. I don’t know*,* I feel like I have more in common with people who have like Hispanic background rather than people who are from like another Multiracial background.” (Participant 11*,* Hispanic & White)*.

Lastly, one participant offered a broader definition of the Multiracial community, shifting the focus away from her personal identity, and instead capturing the nuanced backgrounds that make up this community. This participant described herself as Multigenerational Mixed, or MGM, and explained that individuals in her family have been marrying other Biracial or Multiracial individuals for generations. Her quote demonstrates a deeper level of familiarity and consideration of the Multiracial community:

*“Well*,* Multiracial is very broad*,* so you know*,* there are a lot of different combinations of backgrounds. There’s different cultures. Because*,* like*,* there’s so many mixed race combinations*,* it’s hard to identify like any overlap. But you know*,* there are intersections that can*,* that can be identified.” (Participant 1*,* Black*,* White*,* & Indigenous)*.

### Theme 2: Concerns regarding accuracy of genetic research for multiracial individuals

Participants’ familiarity with genetics research varied widely: five reported no familiarity, one had a basic understanding, three were familiar through personal or family genetic testing, and five had knowledge based on professional experience or education.

When asked their thoughts on the impact of genetics research for Multiracial individuals, many participants expressed concerns about accuracy (*n* = 5). This sentiment was expressed by participants with varying education levels and professional backgrounds. The most frequently cited concern revolved around the accuracy of health risk estimates given Multiracial individuals’ unique backgrounds. More specifically, participants wondered whether their unique ancestral backgrounds might influence their risk of developing conditions that are more common in certain populations. They worried that medical providers may not be able to offer them as comprehensive of a health assessment as they would otherwise provide to individuals from a single racial background. Participants frequently referenced their personal Multiracial identities when expressing this concern, as illustrated by the following quote:

*“It’s a little more complex*,* like*,* I got a mix of Colombian*,* Irish*,* Hungarian*,* and German*,* like*,* who has that? It’s not like an easy… ‘Oh*,* well you’re fully Columbian*,* so we can just tell you what the health probabilities or concerns would be.’” (Participant 9*,* Hispanic & White)*.

Two participants’ concerns about accuracy stemmed from the lack of representation and consideration of diverse backgrounds within research. Specifically, they raised concerns about the ability to recruit and distinguish Multiracial individuals from people who identify with one race. One participant noted the omission of her Multiracial identity in surveys as an example:

*“Maybe not studying people who are Multiracial… a lot of times in research*,* you know*,* like*,* they typically ask you to identify your race. I feel like a lot of times researchers don’t look at Multiracial people. Like they ask you to identify one race*,* or if they have you identify that you’re Multiracial*,* they don’t ask any like*,* follow up questions. They just have you like*,* on surveys. they’ll be like*,* choose whatever racial identity. And then for Multiracial people*,* it’s just “two”. But it doesn’t actually ask what.” (Participant 11*,* Hispanic & White)*.

Finally, one participant expressed skepticism about the ability of current research practices to yield sufficiently powered, and therefore meaningful and actionable, risk assessments. This highlights concerns related to statistical significance and methodology:

*“I’d be surprised if the research is at that level that it’s able to say*,* “Okay*,* because you’re a mix of these two backgrounds*,* therefore*,* your risk is X percent as opposed to… I just don’t think that the sample size of the data that we have out there is large enough to give informed estimates that are meaningfully going to change whether I get screening for a certain condition or not.” (Participant 10*,* Middle Eastern & White)*.

It is important to note that this participant works as a physician and has indicated prior research experience. As such, their perspective may be influenced by their biomedical expertise, which could differ from those without similar professional or academic backgrounds. Nonetheless, it demonstrates the broad range of concerns that exist regarding the accuracy of genetics research involving Multiracial individuals.

### Theme 3: Preferences for engaging with stages of the translational research process

**Fig. 1:**
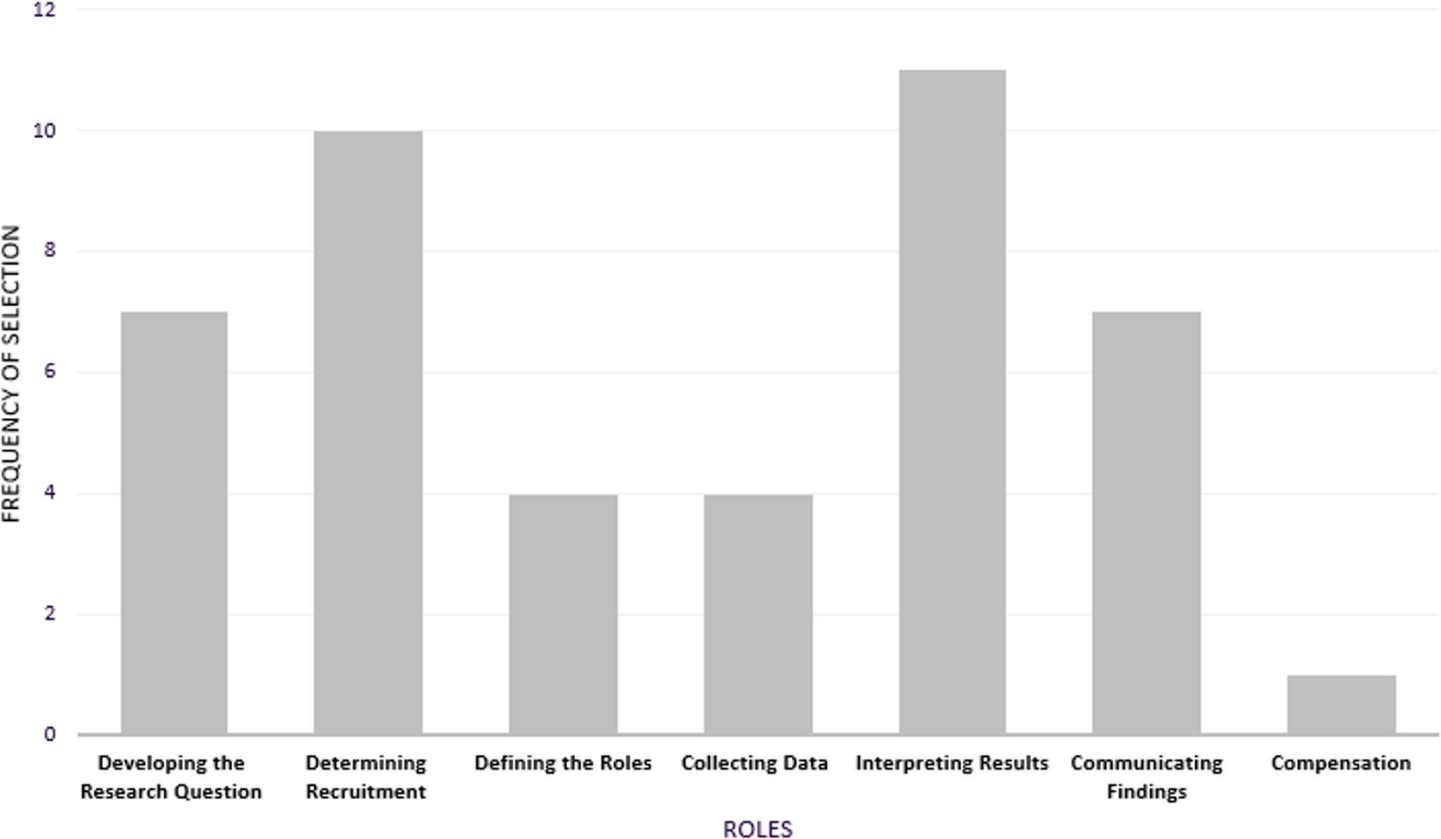
Preferences for engaging with genetics research. Figure 1 is a bar graph titled “Preferences for Engaging with Genetics Research”. It illustrates the frequency of selection among participants for 7 different roles through the genetics research process. ‘Interpreting Results’ and ‘Determining Recruitment’ were selected the most frequently, with frequencies of 11 and 10, respectively. ‘Communicating Findings’ and ‘Developing the Research Question’ were tied for third, with 7 selections each. ‘Defining the Roles’ and ‘Collecting Data’ each had 4 selections, while ‘Compensation’ was selected the least frequent, with only 1 selection

When asked their top three preferences for which stages of the translational research process they would like to be engaged in, participants selected interpreting results the most frequently, followed by determining recruitment. Tied for third place were developing the research question and communicating findings (see Fig. 1).

Participants were asked to explain why they considered certain roles to be more important than others. Most often, participants cited their professional skills, prior experiences, or personal interests as reasons for ranking certain roles above others. *Participants also prioritized roles that they deemed to be critical to the overall research process*,* particularly the interpretation of results*,* as this step leads to the overall conclusions from the study*:

*“…interpreting results*,* because that’s probably the most difficult and the most important in terms of what you did your research on.” (Participant 4*,* White & Asian)*.

Participants’ Multiracial identities explicitly influenced their selection of two roles: determining recruitment and communicating findings. Regarding recruitment, participants explained that Multiracial individuals might have an easier time recruiting others who share their identity, and perhaps even have more creative ideas for recruitment strategies. Several participants emphasized the importance of recruiting a diverse sample of participants to ensure representation of a broad mix of Multiracial identities. One participant specifically noted that their background would enable them to identify gaps that researchers who do not identify as Multiracial might overlook. Additionally, one participant highlighted that her background as a Multiracial individual could help her clarify the research study’s objectives to potential participants and explain the difference between nuanced terms:

*“…especially if you’re trying to explain the difference between race and ethnicity. Like there is a difference between Multiracial and Multiethnic*,* and you could be both*,* but they are two separate things.” (Participant 1*,* Black*,* White*,* & Indigenous)*.

With regard to communicating findings, participants stated that Multiracial individuals might have a greater interest in sharing research findings and perhaps even find it easier to disseminate this information compared to someone who is not part of the community. Participant 11 elaborated on this point by stating the following:

*“…and then communicating findings. I just feel like someone that’s Multicultural is probably gonna have more interest in like sharing their findings with other people who are Multicultural or people who*,* you know*,* fall within that like cultural identity… they’re gonna have an easier time sharing that finding with their community rather than someone that isn’t part of that community.” (Hispanic & White)*.

Lastly, to account for roles that may not have been included in the list provided to participants, they were encouraged to propose additional ways that they could contribute to research. Three participants shared suggestions including: interviewing community members, reviewing the funding and publication process, and receiving clear explanations of the impact that their contributions had on the overall goal of the study.

### Theme 4: Suggestions for recruitment

Participants were asked to draw on both their experiences as Multiracial individuals and their insights into the Multiracial community to offer suggestions for recruiting others from this population. This portion of the conversation specifically focused on identifying suitable locations and important participant characteristics for recruitment. While a subset of participants identified “determining recruitment” as a preferred way to engage in research, this theme reflects input from all participants regarding recommendations for recruiting Multiracial individuals.

Regarding locations, several participants noted that because the Multiracial population represents a younger and growing community, recruiting from neighborhoods and areas that contain a higher proportion of younger individuals, such as cities/urban areas and universities could be effective. Within cities, participants suggested emerging neighborhoods, ethnic neighborhoods (e.g., Chinatown), and cultural events. Regarding universities, participants specifically mentioned Multicultural sororities and organizations/clubs centered around race or ethnicity. Participants justified their suggestions by mentioning past encounters with individuals of Multiracial backgrounds in these areas.

Regarding participant characteristics, several individuals emphasized the importance of including a range of Multiracial identities, beyond those that are more common in the population. Some participants noted that Biracial individuals who identify as half Black and half White are often considered the most typical Multiracial background. They acknowledged that while recruiting individuals from this racial background is important, including individuals with combinations of other racial backgrounds should also be prioritized. Some individuals specifically emphasized the need to include those with a combination of two or more minority races. For example, Participant 14 mentioned the importance of including individuals who are Asian and Black, as this a more “uncommon”, and potentially marginalized Multiracial group.

*“I feel like there’s not only biracial. But there’s like totally like mixed individuals too that are coming from like multiple races. So I would look into that. I think gathering people from like all different realms of the world. Making sure you have some people from all regions.” (Participant 13*,* Asian & White)*.

*“I feel like a lot of Multiracial people will have like half White in them*,* since the majority of people in the US are Caucasian people. So there isn’t really a specific one that*,* if I was talking to a researcher*,* that I would tell them to focus on*,* I’d just try to get everybody in there.” (Participant 12*,* Asian & White)*.

As previously mentioned, participants recognized society’s shift toward a more diverse population with an increasing number of individuals from multiracial backgrounds. They suggested that recruiting from younger and more open-minded demographics could be advantageous for this reason.

## Discussion

This qualitative study is one of the first to elicit the perspectives and preferences of Multiracial individuals when it comes to engaging with stages of the translational genetics research process. This was accomplished by soliciting each participants’ unique viewpoints about participation in genetics research and exploring the concept of the Multiracial community more broadly.

The findings of this study emphasize that the Multiracial community is a unique and rapidly evolving population. When participants were asked to share their thoughts about this community, they had diverse definitions of what it means to be part of a Multiracial community informed by their own personal experiences. Some individuals, particularly those from biracial backgrounds, defined it based on their personal experiences. Others argued that a clearly defined Multiracial community does not exist. This is in comparison to the suggestion that the Multiracial community is an evolving population which came from the participant who identified as Multigenerational Mixed. This individual may have had more discussions with their family about the Multiracial community and thus a more well-defined or developed Multiracial identity. As this population continues to grow, so will the perspectives and experiences of its members, requiring more thoughtfulness from the research community regarding how to incorporate such diversity within one community. These findings mirror the challenges identified by other researchers, specifically the difficulty of categorizing Multiracial individuals (Parker et al. 2015). Altogether, these challenges in categorization may contribute to the exclusion of Multiracial individuals from scientific research, leading to a limited amount of information about what distinguishes the Multiracial community from others. This suggests that moving forward, it may be beneficial to shift away from categorization and instead, adopt a more inclusive approach that considers the diverse range of Multiracial identities within this population.

While participants had differing views on how to characterize the Multiracial community, many shared a similar concern about genetics research involving this population. Participants voiced concerns without receiving any prior background information from researchers during interviews, demonstrating a high degree of consistency with existing limitations in the field of genetics. Specifically, their responses reflected three key issues identified in the literature: the underrepresentation of diverse populations leading to missed genetic variation and reduced clinical utility (Sirugo et al. 2019), the lack of standardized protocols for collecting or estimating diverse backgrounds (Popejoy et al. 2020), and ongoing challenges in accurately accounting for Multiracial individuals in genomics research (Martschenko et al. 2023). Until we address these challenges in accuracy and ensure that genetics research is inclusive and applicable to all individuals, especially those with diverse backgrounds, the full potential of genetics research may remain unrealized.

Altogether, participants’ concerns about accuracy, along with the historical underrepresentation of diverse backgrounds within the field of genetics, highlight the urgent need to include individuals who fall outside the discrete categorization used in genetics research and society. While recommendations guiding community engagement efforts offer a range of ideas for how to engage individuals (Kwon et al. 2017; Lemke et al. 2022; Sanders Thompson et al. 2020), no recommendations have specifically taken the preferences of Multiracial individuals into account. Moreover, the lack of clarity about the nature of the Multiracial community poses additional challenges for how to meaningfully engage this population. The present study reveals that participants’ preferences for engaging with research vary according to their skills and interests. This indicates that providing participants with the option to choose how they would like to be involved in research could lead to more meaningful, strength-based participation. Participants’ top preferences span the research cycle and suggest that involvement should occur across all stages. This aligns with recent recommendations made by community partners in a study exploring community engaged research (Han et al. 2021).

Lastly, participants’ recommendations for researchers to include a wide range of Multiracial identities in their studies align with Charmaraman et al.’s suggestion to approach the study of Multiracial individuals cautiously, avoiding treating them as a monolithic group, and instead, focusing on analyzing distinct subgroups to gain a more accurate understanding (2015). The diverse responses received from participants in this study underscore the fact that Multiracial individuals differ significantly in terms of their unique identities, experiences, and viewpoints. Therefore, generalizing findings to all who identify as Multiracial may not always be accurate or feasible. For this reason, we support our participants’ and Charmaraman et al.’s (2015) suggestions to include a wide array of Multiracial identities and when possible, focus on more specific subgroups of Multiracial identities.

### Study strengths and limitations

As previously stated, most research on Multiracial individuals has been confined to a subset of social science fields, limiting our understanding of how community engagement strategies can be effectively applied within genetics research involving this population. Exacerbating this issue is the fact that the Multiracial population is often overlooked due to its diverse and hard-to-define nature. This study offered much needed insights into Multiracial individuals’ sense of community, as well as their preferences and suggestions for engaging with genetics research. Furthermore, this study informs future community-engaged research studies by proposing recruitment strategies for involving individuals from the Multiracial community based on suggestions from Multiracial individuals themselves.

Two limitations were identified after the conclusion of the study. First, participants were not explicitly asked whether they wanted to engage in the research process. Asking them to rank their top three roles assumed their interest, which might not have been the case. Second, the insights and opinions provided by participants were not always specific to research in genetics, but rather pertained to scientific research more broadly.

Given the exploratory nature of this study, we recommend that future research studies focus on implementing recruitment and engagement strategies to involve Multiracial individuals in genetics research. We stress the importance of carefully considering the recommendations, concerns, and ideas shared by participants in this study, and advocate for the continuous incorporation of community members’ voices to ensure more meaningful and effective forms of engagement moving forward. Additionally, the current study focused specifically on research as it relates to genetics; therefore, future studies could aim to delineate between research and healthcare.

## Conclusion

The projected growth of the Multiracial population signifies that it is now more important than ever to reimagine how individuals from this community are engaged in research to ensure that benefit sharing in genomics serves all populations moving forward (Martschenko et al. 2023). As definitions of the Multiracial community evolve alongside the expected growth of this population, it will be vital to adopt a more creative approach to meaningfully engage Multiracial individuals in genetics research. Researchers interested in engaging members of this community should be flexible with changing definitions, honor participants’ unique needs and preferences, and incorporate the recruitment suggestions expressed by members of this community. Finally, the varying perspectives highlighted within this study demonstrate that as a society, we should avoid rigid categorizations to more accurately capture Multiracial individuals’ backgrounds and experiences.

## Supplementary Information

Below is the link to the electronic supplementary material.

Supplementary Material 1

## Data Availability

No datasets were generated or analysed during the current study.

## Footnotes

1 Some argue that inter-rater reliability aligns qualitative research with quantitative rigor, others contend that it may oversimplify or misrepresent the nuanced, interpretive nature of qualitative analysis. This study aims to navigate this complexity by employing inter-rater reliability as one tool among many to enhance transparency and reflexivity in the analytic process.
