# Supplementary material for: Multiracial individuals’ perspectives on participating in genetics research
Source: J Community Genet. 2026 Jun 5;17(3):71. doi: 10.1007/s12687-026-00902-x (PMC13241369; doi:10.1007/s12687-026-00902-x)
Supplement: Supplementary file 1 — Supplementary Material 1 [file 12687_2026_902_MOESM1_ESM.docx]

**Supplementary Item 1: Interview Guide**

Welcome and thank you for joining us today. As you may recall from the survey that you filled out, this focus group is being conducted to explore the thoughts and perspectives of Multiracial individuals when it comes to being involved in research. I recognize that your awareness or understanding of research in the field of genetics may vary, and that is completely okay! There is no prior experience needed to participate in today’s conversation, we would simply like to create a space for you all to share your thoughts and opinions.

To give you an overview of what today’s focus group session will look like, we will stay in this main room the entire time (i.e., there will not be any break-out groups). The session will last no longer than 90 minutes. We will begin with introductions and then move onto our discussions which will account for most of the time that we spend together. At the end, we will wrap up and share final thoughts.

Please keep your cameras on during our session so that we are able to see one another and ensure active participation. Some quick ground rules: we hope that this focus group will be a safe and accepting environment where you can all share your thoughts and feelings. Please be conscientious of not interrupting other people while they are speaking. I encourage you all to use the “raise hand” feature on Zoom if there are multiple people who would like to speak. This will help us make sure that everyone has an opportunity to talk when they would like to! Please feel free to use the chat to communicate as well - questions are always welcome.

There are a few technical and logistical aspects that I would like to discuss with you all before we get started. As you read in the consent form, today’s session will be recorded. The reason why our session is being recorded today is because it will help us translate your thoughts and feelings into data that will eventually be used to write a research paper. More specifically, we will be transcribing the audio recordings from this session. I would like to note that the transcript will be de-identified and you will only be referred to by your participant ID number.

Does anyone have any questions or comments?

[Pause to answer any questions]

In that case, let’s go ahead and begin with introductions! We would appreciate it if you could all share your name, pronouns, racial identity, where you are joining from, and how you describe your Multiracial identity or your Multiracial family.

I’ll go ahead and start calling names.

[All participants will introduce themselves]

Thank you all for introducing yourselves! It was great to hear all of your responses and learn more about your backgrounds.

Now I would like to think about things more broadly and explore the Multiracial community. I would like to learn more about how you all view community. Please take a moment to read the question on the screen, reflect on it and then we will have a discussion.

Main Question:

- When you think about the Multiracial community, what comes to mind?

Probing Questions:

- Do you consider yourself to be a part of this community?
- Are there other racial/ethnic/cultural communities that you identify with more strongly?
  - What are they?
- Does your identity change depending on where you are or who you are around? How?
- How has your multiracial identity changed over time? Does the context that you are in influence your identity? If so, what factors play a role?
- Where do you find yourself connecting with the multiracial community the most?

Additional Probing Questions:

- Can you tell me more about ___?
- Has anyone else felt/experienced something similar?
- Could you share some examples of ____?

I appreciate you all opening up and sharing your thoughts.

I will now share the next question(s) with you all. Please take a few moments to reflect on any ideas that come to mind. We will come back together shortly.

Main Question (EC):

- How familiar are you with genetics research and its potential implications on healthcare?

Main Questions (JY):

- Has your multiracial identity been relevant to your health or healthcare over the course of your life?
  - If yes, how? Please provide examples.
- Has a healthcare provider ever discussed specific health risks related to your racial/ethnic identity?
  - If yes, how? Please provide examples.
- Do you think that your multiracial identity is important for your provider to know about?
  - Why or why not?

Additional Probing Questions:

- Can you tell me more about ___?
- Has anyone else felt/experienced something similar?
- Could you share some examples of ____?

Thank you all for sharing your experiences!

Now we will jump into a brief overview of the research process to gain a better understanding of how genetics research is conducted. As you can see on the screen, the research process can be broken down into five main steps: 1. A concern or gap in knowledge is identified, 2. A plan to collect more information is made. 3. Data is collected. 4. The data is interpreted, and 5. Results are shared. In order to understand this process more clearly, we would like to share an example with you.

Example of Genetics Research:

*During the early 2000s, it was noted that many children in Southern Brazil were being diagnosed with adrenocortical carcinoma, or ACC. This is a very rare cancer that affects hormone secretion, potentially causing patients to develop masculine traits, regardless of the sex they were assigned at birth. This type of cancer can spread to other parts of the body.*

*Since it is such a rare and severe form of cancer, scientists wondered why it was being seen so frequently among children. In 2001, Brazilian families were recruited for genetic testing in order to determine whether there was a genetic explanation for what they were observing.*

*They found that all of the children who developed ACC had a genetic change, or variant, in the gene TP53. This is a very important gene in the body because it prevents cells from growing too quickly, or in other words, turning into cancer. The genetic variant that was identified in the Brazilian children disrupted this control system, therefore leading to the development of cancer.*

*Additional studies confirmed that this variant is very common in the Brazilian community. This led to the development of oncogenetics practice centers in Southern Brazil. Because of this targeted study on Brazilian children, physicians and genetic counselors are now better informed about this unique variant and able to provide the highest level of care to individuals from this community.*

Source: Achatz, Maria Isabel, and Gerard P Zambetti. “The Inherited p53 Mutation in the Brazilian Population.” *Cold Spring Harbor perspectives in medicine* vol. 6,12 a026195. 1 Dec. 2016, doi:10.1101/cshperspect.a026195

Follow-Up Questions:

- In your opinion, what are the potential benefits of genetics research for individuals from Multiracial backgrounds?
- What concerns, if any, do you have regarding genetics research and its impact on individuals from diverse racial backgrounds?

Now that we have reviewed an example, we can revisit the research process. This time, considering roles that community members such as yourselves could undertake as a way to be involved in the genetics research process.

I would like to ask you all to complete an activity: please rank the following roles from 1 to 8.

Roles:

- Developing the research question/research goals
- Determining recruitment (e.g., who will be recruited, how will they be recruited)
- Deciding how participants will be compensated for their time and effort
- Defining the role that community partners will play throughout the research process
- Collecting data
- Interpreting results / the meaning of the data in the context of the broader community
- Communicating findings
- Evaluating the overall efforts to engage community members

Your ranking will reflect where along the research process you believe someone from the Multiracial community would prefer to be involved, with 1 being the most important and 10 being the least. There is no right or wrong answer and the rankings will be anonymous so please feel free to share your honest opinion! I will give you all some time now to complete this activity.

Thank you all for taking the time to complete your rankings! Let’s take a look at your responses and reflect on them.

Main Question: Please explain why you chose to rank the roles in the order that you did and/or

reflect on any differences/similarities that you observe from the group's responses as a whole.

Probing Questions:

- How did you decide to rank each role?
- Why is your top choice the most important to you?
- Are there any other ways that you would like to be involved in genetics research that were not mentioned?

Thank you for volunteering your thoughts! That wraps up our session. I would like to express how grateful I am for your participation today. Hearing your experiences and opinions was incredibly informative. Please reach out to us with any questions or concerns! We will be in touch with you when your gift cards are mailed. Bye, thank you again!
